# Circular Permutation of the *E. coli* Heat-Labile Enterotoxin Pentameric B Subunit for Mucosal Vaccine Adjuvant Design

**DOI:** 10.1021/acsomega.5c12660

**Published:** 2026-01-25

**Authors:** Sheng-Han Hsu, Da-You Xie, Cheng-Yen Tsai, Wei-Shuo Lin, Chu-Ya Wu, Yi-Hung Lin, Wen-Chun Liu, Suh-Chin Wu, Shih-Che Sue

**Affiliations:** † Institute of Bioinformatics and Structural Biology, 34881 National Tsing Hua University, 101, Section 2, Kuang-Fu Road, Hsinchu 300044, Taiwan; ‡ Institute of Biotechnology, National Tsing Hua University, 101, Section 2, Kuang-Fu Road, Hsinchu 300044, Taiwan; § Instrumentation Center, National Tsing Hua University, 101, Section 2, Kuang-Fu Road, Hsinchu 300044, Taiwan; ∥ 57815 National Synchrotron Radiation Research Center, 101 Hsin-Ann Road, Hsinchu Science Park, Hsinchu 300092, Taiwan; ⊥ Biomedical Translation Research Center, Academia Sinica, 128 Section 2, Academia Road, Nankang, Taipei 115201, Taiwan

## Abstract

The heat-labile enterotoxins (LTs) of *Escherichia coli* structurally belong to the AB_5_ toxin, which consists of an A subunit and a pentameric B subunit. LTs have been extensively reported for their potent mucosal adjuvant activities. The B subunit of type II LT (LT-IIb-B_5_) recognizes the GD1a ganglioside, which is associated with the internalization of the toxic A subunit and meanwhile involved in the recognition of the Toll-like receptor (TLR-2/1) and downstream NF-κB activation. In developing LT-IIb-B_5_ as a safe adjuvant, we intend to prevent concerns derived from the intrinsic toxicity of LT. A strategy of circular permutation (CP) is used to design a new LT-IIb-B_5_ pentamer. By replacing the backbone opening at the GD1a-binding site, the GD1a-binding site becomes deficient and GD1a-binding ability is removed, yet TLR-2/1 activation is maintained. The novel LT-IIb-B_5_ pentamer has no GD1a-mediated cell toxicity but maintains sufficient immunoreactivity. We identified a potent CP candidate (CP_13–14_) with a backbone opening at position 13–14, exhibiting greater protective potential against an influenza viral challenge than the native LT-IIb-B_5_ in a mouse model. In the intranasal vaccination using trivalent neuraminidase proteins formulated with LT-IIb-B_5_ CP_13–14_, we demonstrated complete protection against heterologous H1N1 and H3N2 viral challenge infections. This study purposes for a new design for LT-IIb-B_5_ protein adjuvant and addresses the urgent need for a novel, efficient, and safer mucosal adjuvant for respiratory diseases.

## Graphical abstract

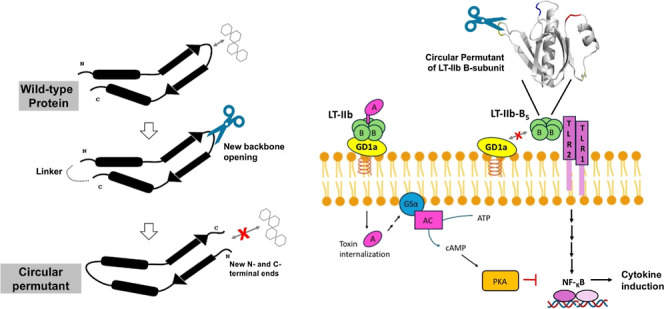

## Introduction

The mucosal system appears to be the major target of most human pathogens.^1,2^ The infection might be prevented by a unique, potent pre-existing immune response in mucosal compartments. However, the major constraint in the development of mucosal vaccines is that antigens applied to mucosal membranes generally induce relatively weak immune responses, which is to avoid inducing severe responses to the numerous harmless antigens in the environment.^2,3^ To overcome the barrier, mucosal adjuvants must be employed to elevate the immune response. The incorporation of an appropriate adjuvant in vaccine formulations will assist in the stronger induction of protective immunity.

*Vibrio cholerae toxin|cholera toxin* (CT) and *Escherichia coli* heat-labile enterotoxins (LTs) belong to a structurally similar protein family, and LTs have been extensively reported for their potent adjuvant activities. The family is classified into type I (LT-I) and type II (LT-II) subfamilies, depending on their antigenic capabilities and related genetic sequences.^4^ Each of these enterotoxins has a hexameric AB_5_ structure, consisting of an A subunit with catalytic activity and a pentameric B subunit responsible for binding to host glycoconjugates. The A subunit is an enzyme that catalyzes ADP-ribosylation of the G_sα_ regulatory protein, which upregulates adenylyl cyclase. The regulation of G_sα_ enhances the intracellular adenosine 3′,5′ cyclic monophosphate (cAMP) level.^5,6^ The B pentamer is involved in the binding of gangliosides, a diverse family of glycolipids found on mammalian cell surfaces.^7^ The two LT subfamilies have different binding pattern to one or more gangliosides.^4^ The B subunit is not toxic, but the affinity for gangliosides facilitates the uptake of the entire toxin by host cells through receptor-mediated endocytosis, enabling the A subunit to translocate into the cell cytoplasm. The modification leads to persistent activation of adenylate cyclase, which elevates cAMP levels. Increased cAMP activates protein kinase A, which phosphorylates the cystic fibrosis transmembrane conductance regulator (CFTR) chloride channels on the apical surface of intestinal epithelial cells. This activation results in an increased secretion of chloride ions and water into the intestinal lumen, while sodium absorption is inhibited, exacerbating fluid loss and leading to severe watery diarrhea.^8,9^

Members of the LT-II subfamilies have been shown to be effective mucosal adjuvants in numbers of studies.^4,6,10^ It was reported that the noncatalytic B subunits of Type II enterotoxins interact with Toll-like receptors (TLR). In a TLR-2 signaling pathway, LT-IIa-B_5_ and LT-IIb-B_5_ were demonstrated to activate human monocytes and mouse macrophages. TLR-1, which heterodimerizes with TLR-2, was also found to be stimulated by LT-IIa-B_5_ and LT-IIb-B_5_.^11^ LT-IIb holotoxin does not bind or activate the TLR-2/1 heterodimer due to the steric hindrance relate to the A subunit.^12^ In the absence of an A subunit, LT-IIb-B_5_ initially binds the lipid raft-associated GD1a ganglioside, which recruited the TLR-2/1 signaling complex to lipid rafts. This GD1a-binding might facilitate the interaction of LT-IIb-B_5_ with the TLR-2/1 signaling complex, activating the inflammatory transcription factor NF-KB through the TLR adaptor proteins MYD88 and TRIF. A consequence of NF-KB activation is the production of inflammatory cytokines such as TNF-α, IL-1β, IL-6, and IL-8.^4,13^ This mechanism implies crucial connections with the innate immune system, thus, identifying LT-IIb-B_5_ as a possible immunomodulatory adjuvant.^4,13–15^

For the 2000–2001 influenza season, an approved inactivated virosomal-subunit influenza vaccine (Nasalflu, Berna Biotech), which contained *E. coli* LT as a mucosal adjuvant, was available in Switzerland. Unfortunately, following emerging cases of Bell’s palsy, this vaccine is no longer for clinical usage.^16^ The following investigation suggests an association between Bell’s palsy and the inactivated intranasal influenza vaccine. Although the etiology and pathogenesis of Bell’s palsy still remain to be adequately studied, the increased risk of Bell’s palsy after vaccination may be related to vaccine components.^16–19^ LT-IIb-B_5_ has a high affinity for GD1a. This binding allows the entire toxin to enter cells and exert its toxic effects. Previous studies have shown that the CT has a similar mechanism and AB_5_ structure that the side effects caused by LT could be due to the process with toxin internalization and ganglioside binding.^8,10,20,21^ CT induced proinflammatory responses when administered intranasally.^21^ In a mouse model, intranasally administered CT was transported from the nasal mucosa to the olfactory bulbs, impairing odor responses in the olfactory system.^22^ However, the B_5_ unit of CT alone did not damage the olfactory system,^22^ and a CT mutant that cannot bind to monosialoganglioside (GM-1) did not accumulate in the nervous system.^23^ Thus, removing GD1a binding could eliminate the toxicity and modulate the immunoreactivity of LT-IIb-B_5_. Based on this understanding, designing an LT-IIb-B_5_ mutant that cannot bind to GD1a will be critical to using it as a vaccine component.

Previous reports show that Thr13 and Thr14 in LT-IIb-B_5_ were the critical residues for GD1a binding.^4,24–26^ The Thr13 and Thr14 mutations in LT-IIb-B_5_ lead to a decreasing binding ability to GD1a.^9,27^ Moreover, the crystal complex structures of LT-IIb-B_5_ and Neu5Ac-nLT, the core chain of ganglioside, reported the sialic-binding residues consisting of Arg12, Thr13, Thr14, Ile30, Asn31, Asn32, and Trp92. The crystal structure shows that four of the ganglioside ligands bind in a groove between position 12–16 of one monomer and position 29–36 of the neighboring monomer. In addition to the four ligands, there is the fifth ganglioside molecule found in one monomer, where it interacts with the residues Arg51, Lys53, Asp54, and Tyr55, providing a possible secondary GD1a-binding site.

In the present study, we adopted the strategy of protein circular permutations|circular permutants (CPs) to design the new LT-IIb-B_5_, in which the original N- and C-termini are connected, and the new backbone N- and C-terminal ends are relocated. We designed LT-IIb-B_5_ CPs and evaluated the structural properties and GD1a binding. We intended to identify CP candidates with good structural stability, eliminated GD1a-binding ability, and comparable TLR-2/1 activation. These features could make the new LT-IIb-B_5_ a better substitute for native LT-IIb-B_5_ in developing a mucosal adjuvant.

We tested LT-IIb-B_5_ CPs on a mouse model and compared the results to those of native LT-IIb-B_5_. We used influenza virus neuraminidase (NA) proteins as antigens along with the LT-IIb-B_5_ CP protein adjuvant to formulate a nasal spray vaccine. Of the tested CPs, one candidate, CP_13–14_, exhibited a greater protective potential against an influenza viral challenge. This study may contribute to the development of an effective and safe mucosal adjuvant in the future.

## Experimental Section

### Preparation of LT-IIb-B_5_ Circular Permutations

We designed LT-IIb-B_5_ CPs, which cleavage position 13–14, 31–32, and 52–53 (named CP_13–14_, CP_31–32_, and CP_52–53_, respectively) into new N- and C- termini. A four-residue (GSGS) linker was added to connect the original N- and C- termini. LT-IIb-B_5_ CP genes had been synthesized and inserted into vector pET22b (+) plasmid with a C-terminal His tag for aiding purification. *E. coli* BL21­(DE3) (Invitrogen) was used for protein expression. The transformation was performed by the heat-shock method and subsequently spread on the agar plates. After incubating overnight at 37 °C, a single bacterial colony was picked to incubate in 20 mL Luria–Bertani (LB) medium and cultured overnight at 37 °C. Cells were then transferred and cultured in 1L LB medium with vigorous shaking at 37 °C. When OD_600_ reached 0.6, the cells were induced by 1 mM isopropyl β-d-1-thiogalactopyranoside (IPTG) for 20 h at 16 °C. The cells were harvested. The cell pellet was resuspended in sample buffer (50 mM Tris–HCl pH 7.0 and 150 mM NaCl) and subsequently lysed by a high-pressure homogenizer. Separating the insoluble fraction that contains LT-IIb-B_5_ by centrifugation at 15000*g* for 30 min, a standard protein dialysis process was performed to refold LT-IIb-B_5_ proteins. The supernatant containing folded LT-IIb-B_5_ proteins was purified by Ni-charged IMAC resin (GE Healthcare). The nonspecific binding proteins were removed by the washing buffer (50 mM Tris–HCl pH 7.0, 150 mM NaCl, and 40 mM imidazole), and the bound His-tagged LT-IIb-B_5_ proteins were eluted with the elution buffer (50 mM Tris–HCl pH 7.0, 150 mM NaCl, and 400 mM imidazole). We concentrated LT-IIb-B_5_ proteins by using an Amicon membrane ultrafilter (Millipore). Gel filtration chromatography was performed to optimize the purity. A HiLoad 16/60 Superdex-75 column (GE Healthcare Life Sciences) was equilibrated with the sample buffer before injection and under the control of an ÄKTA-FPLC system controller. For mouse immunization, the residual lipopolysaccharides in LT-IIb-B_5_ samples were removed by an endotoxin removal spin column and confirmed by an LPS assay kit (ThermoFisher Scientific). To prepare the uniformly ^15^N-labeled protein for the NMR study, the M9 medium was supplemented with ^15^NH_4_Cl (1 g/L) as the sole nitrogen source.

### Circular Dichroism

CD spectra were examined on the J815, Jasco CD spectrometer. LT-IIb-B_5_ and CPs were investigated at the concentration of 8 μM using a 0.1 cm path-length quartz cuvette (Starna Scientific, UK) with the protein buffer of 5 mM phosphate buffer, pH 7.0, and 50 mM NaCl. We recorded a CD spectrum at every 10 °C increment that the machine increased the temperature from 10 to 90 °C. The measurement spans three repeat spectral runs, from 260 to 190 nm. By using the optical path length and protein content as inputs, molar ellipticity was calculated and converted from the unit of mdeg.^28^

### Differential Scanning Fluorometry

We used the Tycho NT.6 machine (Nanotemper, from the HSU CH lab, NTU) to conduct differential scanning fluorometry experiments. We prepared 10 μL of samples at concentrations ranging from 1.5–2 mg/mL with the buffer of 20 mM phosphate buffer, pH 7.4, and 150 mM NaCl. Samples were drawn into capillaries and inserted into the machine. The machine gradually increased the temperature from 35 to 95 °C at a temperature rate of 1 °C/min. The machine detected the wavelength at 330 and 350 nm. The recorded wavelength ratio (350 nm/330 nm), representing the change in tryptophan fluorescence intensity, was plotted as a function of temperature.^28,29^

### NMR HSQC Spectroscopy and Titration Experiment

Proteins were purified and then transferred to an NMR buffer (50 mM Tris, pH 6.0, and 10% D2O). The final samples were transferred into Shigemi NMR tubes, and the measurements were performed on Bruker 600 or 850 MHz spectrometers. NMR data were referenced to the ^1^H resonance frequency of 2.2-dimethyl-2-siapentane-5-sulfate (DSS) at 0 ppm. A one-dimensional NMR titration experiment was performed using the unlabeled LT-IIb-B_5_ and GD1a samples. The 1D ^1^H spectrum of the free LT-IIb-B_5_ sample and free GD1a were acquired at 298 K. GD1a was added incrementally to achieve titration ratios of 1:1, 1:2, and 1:3. The respective 1D ^1^H spectrum was recorded at each titration step. The 2D titration experiment was performed using the ^15^N-labeled LT-IIb-B_5_ sample and the unlabeled GD1a sample. The ^1^H–^15^N HSQC spectrum was used to detect the binding between LT-IIb-B_5_ and GD1a.

### X-ray Data Collection, Processing, and Determination

CP_13–14_ and CP_52–53_ proteins were concentrated at 3 mg/mL in 50 mM Tris–HCl, pH 7.0, and 150 mM NaCl and crystallized by the hanging drop vapor diffusion method. The precipitation conditions were screened by Kit in 48-well crystal growth plates. Single crystals were transferred to the fomblin for X-ray diffraction experiments. CP_13–14_ and CP_52–53_ X-ray diffraction data were collected at beamline BL05A and BL13B1 equipped with an ADSC Quantum-315r CCD Area Detector in the National Synchrotron Radiation Research Center. Using the application HKL2000, data sets were indexed, integrated, and scaled. The structures of CP_13–14_ and CP_52–53_ were determined by molecular replacement with Phaser-MR (Phenix supported program) using the published structure of LT-IIb-B_5_ (PDB 1QB5) as the search model. After refinement and simulated annealing using Phinix.refine, several rounds of manual model building in COOT were performed to improve the quality and completeness of the structure. The structures of CP_13–14_ and CP_52–53_ were deposited in the protein data bank with codes of 8GW2 and 8H2R.

### TLR-2/1 (hTLR-2/1) Activity

HEK 293A cells were genetically transfected to overexpress human TLR-2/1 (hTLR-2/1) heterodimer and NF-kB-driven luciferase (InvivoGen, USA). The cells were treated with LT-IIb-B_5_ and incubated for 5 h at 37 °C. The binding between LT-IIb-B_5_ and hTLR-2/1 triggers the intracellular signaling pathway to activate the NF-κB promoter, leading to the expression of the downstream luciferase gene. The luciferase activity is measured by adding luciferase substrate, and the result is used to represent the activity of LT-IIb-B_5_ toward activated TLR-2/1. Pam3CSK4 (InvivoGen) is a synthetic lipopeptide that activates the TLR-2/1, which is used as a positive control.

### Mouse Immunization

Female BALB/c mice aged 6 to 8 weeks were purchased from the National Laboratory Animal Center, Taiwan. These mice were intranasally immunized with recombinant influenza virus NA from virus strains of H1N1 (A/California//07/2009), H3N2 (A/Udorn/307/1972), and IBV (B/Darwin/07/2019) along with 10 μg of the recombinant LT-IIb-B_5_ proteins (adjuvant) or PBS as control, where N1NA (N1*) contains two additional glycan-masking mutations of N329T and K331T to enhance the cross-reactivity of different strains of virus. Prior to intranasal administrations, mice were under anesthetization and then administered a 30 μL injection containing NAs (with or without LT-IIb-B_5_). All groups of mice were immunized three times at weeks 0, 3, and 6. Sera were sampled at week 8. These mice were euthanized at week 9 for the collection of bronchoalveolar lavage fluid (BALF). All animal procedures were conducted in compliance with the guidelines established by the Laboratory Animal Center of National Tsing Hua University (NTHU) and were reviewed and approved by the NTHU Institutional Animal Care and Use Committee (approval no. 10246).

### ELISA Assay of Antibody Titers

The procedure regarding the analysis of NA-specific antibody titers is as follows. ELISAs were utilized to quantify antibodies in sera and BALF samples from immunized mice. The 96-well ELISA plates were coated with 100 μL (2 μg/mL) of recombinant NA protein and immobilized at 4 °C for 16 to 18 h. Subsequently, the plates were blocked with blocking buffer (1% BSA in PBS) at 37 °C for 2 h. Serial dilutions of sera or BALF samples were added to each plate and incubated for 1 h at room temperature. Following this, HRP-conjugated goat antimouse IgG antibodies (1:30,000) or HRP-conjugated goat antimouse IgA antibodies (1:50,000) were added to the wells and incubated for an additional 1 h at 37 °C. TMB Chromogen Solution (BioLegend) was mixed for color development, followed by incubation for 15 min at 37 °C. The reactions were then halted with 2 N H_2_SO_4_ and measured by an ELISA reader (OD_450_).

### Neuraminidase Inhibition Assay

ELISA plates coated with 100 μL (50 μg/mL) of fetuin (Sigma) were incubated at 4 °C overnight. Then, the plates were washed three times with PBST buffer and blocked with PBST buffer for 2 h. Viruses with OD_450_ value of 2 measured by ELISA assay were coincubated with equal volumes of 2-fold serially diluted sera samples for 1 h at 37 °C and transferred to ELISA plates coated with fetuin for 1 h at 37 °C. After three washes with PBST buffer, 100 μL (2.5 μg/mL) of lectin (Sigma) was added. After 1 h incubation at room temperature and three washes with PBST buffer, TMB Chromogen Solution was added to the plates and incubated for 15 min in dark. The reactions were terminated by 2N H_2_SO_4_. The OD_450_ signal was detected by an ELISA reader. The dilutions that inhibited 50% of the NA enzyme activity were defined as IC_50_ values.

### Replication Inhibition Assay

To determine the virus replication inhibition efficacy of sera, a replication inhibition assay was performed. Briefly, pooled sera were pretreated with 3 volumes of receptor-destroying enzyme (Deben, UK, 370,013) at 37 °C for 16–18 h, heat-inactivated at 56 °C for 30 min. The diluted sera were 2-fold serially diluted in MEM-α supplemented with 0.5 μg/mL trypsin-TPCK, mixed with an equal volume of diluted virus (A/California/07/2009 (H1N1), A/Udorn/307/1972­(H3N2), and B/Darwin/07/2019 (IBV)), and incubated at 37 °C for 2 h. After washes with PBS, the preseeding 3 × 10^4^/well MDCK cells were inoculated with sera-virus mixtures at 37 °C for 3 days. The end point of replication inhibition was measured by using the hemagglutination assay.

### Virus Challenges

All mice were housed in AAALAC accredited Animal Biosafety Level 2 (ABSL-2) laboratory of Infectious Disease Core facility, Biomedical Translation Research Center (BioTReC), Academia Sinica. All virus challenge procedures in this study were performed in accordance with the animal protocol (BioTReC-112-D-018) approved by the Institution Animal Care and Use Committee of BioTReC. Female BALB/c mice aged 6–8 weeks were intranasally inoculated with either three doses of a subunit monovalent or trivalent vaccine along with protein adjuvant CP_13–14_ or a control of PBS. At 3 weeks post-third vaccination, the immunized mice were intranasally exposed to the H1N1 (A/Puerto Rico/08/1934) or H3N2 (A/Aichi/2/1968) influenza virus at a 5-fold mouse median lethal dose (LD50) (5x LD_50_). The survival rates and body weights of the mice were monitored and recorded daily for a continuous period of 2 weeks. The individual mouse with body weight loss of 25% or more was considered as the humane end point and euthanized.

### Statistical Analyses

All results were analyzed using GraphPad Prism v6.01 software. Antibody titers among more than two comparable groups (excluding the PBS control) were analyzed using one-way analysis of variance (ANOVA) with Tukey’s multiple comparisons tests. Statistical significance in all results is indicated as follows: *p < 0.05 and **p < 0.01. The IC-50 NAI titers were obtained by fitting the dose-dependent curves from pooled sera from each immunized group using GraphPad Prism v6.01. The survival curves were compared using the log-rank (Mantel–Cox) test. Statistical significance was denoted as follows: *p < 0.05, **p < 0.01, **p < 0.001, and **p < 0.0001.

## Results

### Design of LT-IIb-B_5_ Circular Permutants

The primary GD1a-binding site consists of residues Arg12, Thr13, Thr14, Ile30, Asn31, Asn32, and Trp92. Arg12, Thr13, and Thr14 are located in the loop of residues 12–16, while Ile30, Asn31, and Asn32 are located in the loop of residues 29–36 (Figure 1A). In addition, residues Arg51, Ala52, and Lys53 have been speculated to be important for the secondary GD1a-binding site. These residues are located in the loop of residues 50–55 (Figure 1A). As observed in the X-ray structure, these loops contain structural flexibility. To diminish the GD1a binding affinity of LT-IIb-B_5_, we designed LT-IIb-B_5_ CPs that the loops involved in GD1a-binding site are cleaved to disrupt the local structure. The introduced cleavages created a GD1a-binding deficient mutant. We selected the CP sites in the flexible loops and intended to do no harm to the protein fold. To quantitatively evaluate the best sites for creating a viable CP, a Web site-based predictor, CPred, was used to visually inspect which residue will be the best site for being a viable CP site.^30^ The prediction outputs a score (0 to 1) for each residue. We selected the positions with the highest scores. In the primary GD1a-binding site, positions 13 and 14 with the local highest score of 0.99 and positions 31 and 32 of score 0.95 were selected that correspond to the constructs of CP_13–14_ and CP_31–32_, respectively (Figure S1). Noticeable, another critical residue Trp92 is located on the region with secondary structure, principally inapplicable for being a CP site. On the other hand, for the secondary GD1a-binding site, position 52–53 of score 0.94 was selected. The corresponding construct is CP_52–53._ We designed three LT-IIb-B_5_ CPs that disconnected positions 13–14, 31–32, and 52–53, creating new N- and C- termini (Figure 1B). The distance between the original N- and C-termini is approximately 8 Å, and a four-residue (GSGS) linker was added to connect them. These CP sequences are constructed into an expression vector (Figures 1C and S2).

**Figure 1:**
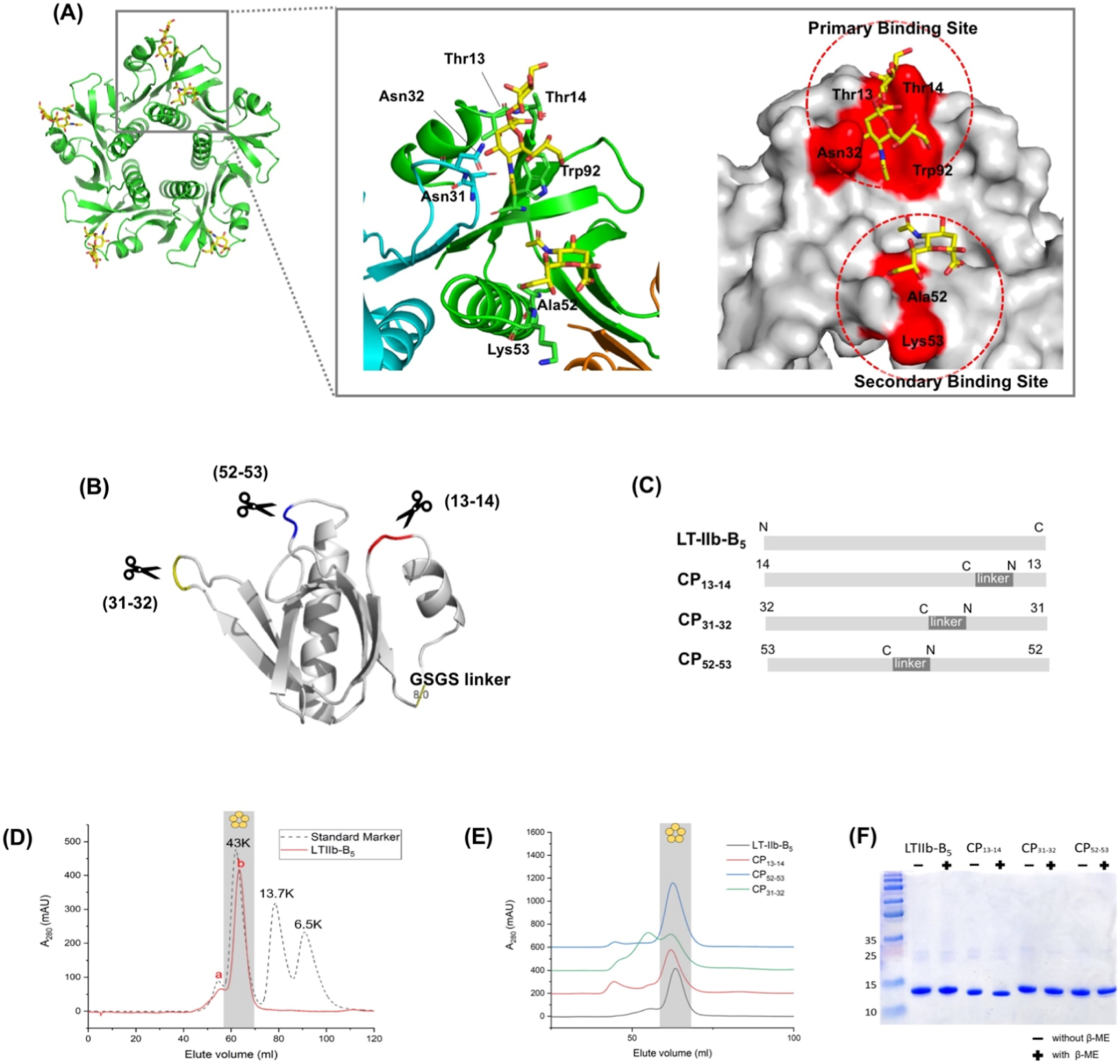
Design of LT-IIb-B_5_ CP constructs. (A) The structure of the LT-IIb-B_5_ pentamer in complex with GD1a (PDB 5G3L). There are predicted primary and secondary GD1a-binding sites on the LT-IIb-B_5_ surface. The respective critical residues responsible for binding are indicated. (B) The selected CP sites (13–14, 31–32, 52–53) are indicated on the LT-IIb-B_5_ monomer. The three CPs of CP_13–14_, CP_31–32_, and CP_52–53_ are designed by respectively disconnecting the CP sites and connecting the original terminal ends by a GSGS linker. (C) The backbone rearrangement of CP_13–14_, CP_31–32_, CP_52–53_ are depicted. (D) HiLoad 16/60 Superdex-75 size exclusion column chromatography of LT-IIb-B_5_. Gel Filtration Calibration Kits (LMW) were used as the standard markers. Peak b represents pentameric LT-IIb-B_5_ and peak a represents aggregated LT-IIb-B_5_. (E) Size exclusion chromatography comparison of LT-IIb-B_5_ and the CPs. The shaded area represents the pentameric molecular weight of LT-IIb-B_5_. (F) SDS-PAGE analysis of purified LT-IIb-B_5_ and the CPs in the presence and absence of β-mercaptoethanol (β-ME).

### LT-IIb-B_5_ Pentamer

The expressed CPs are with a signal sequence, pelB leader, in the N-terminus and a His-tag in the C-terminus. The pelB leader sequence led to the protein secreted to the bacterial periplasm, where the leader sequence was spontaneously cleaved and separated from the LT-IIb-B_5_ sequences. The His-tag in the C-terminus is used to facilitate the purification by a Ni-column. After Ni-column purification, we collected the elution fraction and applied into FPLC size-exclusion column. LT-IIb-B_5_ was primarily distributed at an elution volume of 60–65 mL (Figure 1D, peak b), which was close to the elution volume of the 43 kDa protein standard. A small amount of aggregated LT-IIb-B_5_ was seen to elute earlier (Figure 1D, peak a). The LT-IIb-B_5_ monomer has a molecular size of 10 kDa. The elution volume matched the observation of the LT-IIb-B_5_ pentamer (50 kDa). We further evaluated the CPs. The three CPs had similar elution volumes, indicating the same pentamer state (Figure 1E). The relocation of N- and C- termini to positions 13–14, 31–32, and 52–53 did not change the pentameric formation. Noticeable, more protein aggregation was observed in the CP_31–32_ purification (Figure 1E). In SDS-PAGE assay, the LT-IIb-B_5_ had a denatured monomeric state with an apparent molecular size of ∼12 kDa (Figure 1F). Since LT-IIb-B5 crystal structure shows an internal disulfide bond between two cystine residues, Cys10 and Cys81,^31^ we checked the samples in the absence of β-ME to detect the presence of the disulfide-bond linked dimer. In the conditions, a similar monomer pattern was observed in the SDS-PAGE, indicating the absence of an intermolecular disulfide bond (Figure 1F). This result confirmed no disulfide bond-linked dimer existed between the two LT-IIb-B_5_ protomers.

### The Structural Stability

CD spectroscopy was used to monitor changes in the secondary structure during continuous temperature gradient experiments. As the temperature increases, the CD spectra of LT-IIb-B_5_ show significant changes that may indicate pentamer dissociation and structural denaturation. The molar ellipticity at 200 nm dramatically decreased to more negative values as the temperature increased (Figure 2A). Compared with native LT-IIb-B_5_, the secondary structure composition of each CP showed no significant difference (Figure 2A). These results indicate that CPs do not affect the folding of LT-IIb-B_5_. As the temperature increased, we observed a similar change in CD, again reflecting the structural similarity. To assess the structural stability of different CPs, we performed Nano-DSF experiments to determine the melting temperature. As the temperature increased, the proteins underwent thermal denaturation, exposing intrinsic tryptophan residues Trp47 and Trp92. The experiment measured the absorbance ratio of tryptophan (350 nm/330 nm) (Figure 2B, left panel). Calculating the first derivative of the profiles (Figure 2B, right panel) revealed that native LT-IIb-B_5_ had the highest *T*_m_ (90.78 °C), while CP_13–14_ had a slightly lower *T*_m_ (87.33 °C), and CP_31–32_ and CP_52–53_ had an even lower *T*_m_ (83.96 and 83.48 °C, respectively). These *T*_m_ values did not differ significantly, demonstrating that the CPs maintained the protein stability.

**Figure 2:**
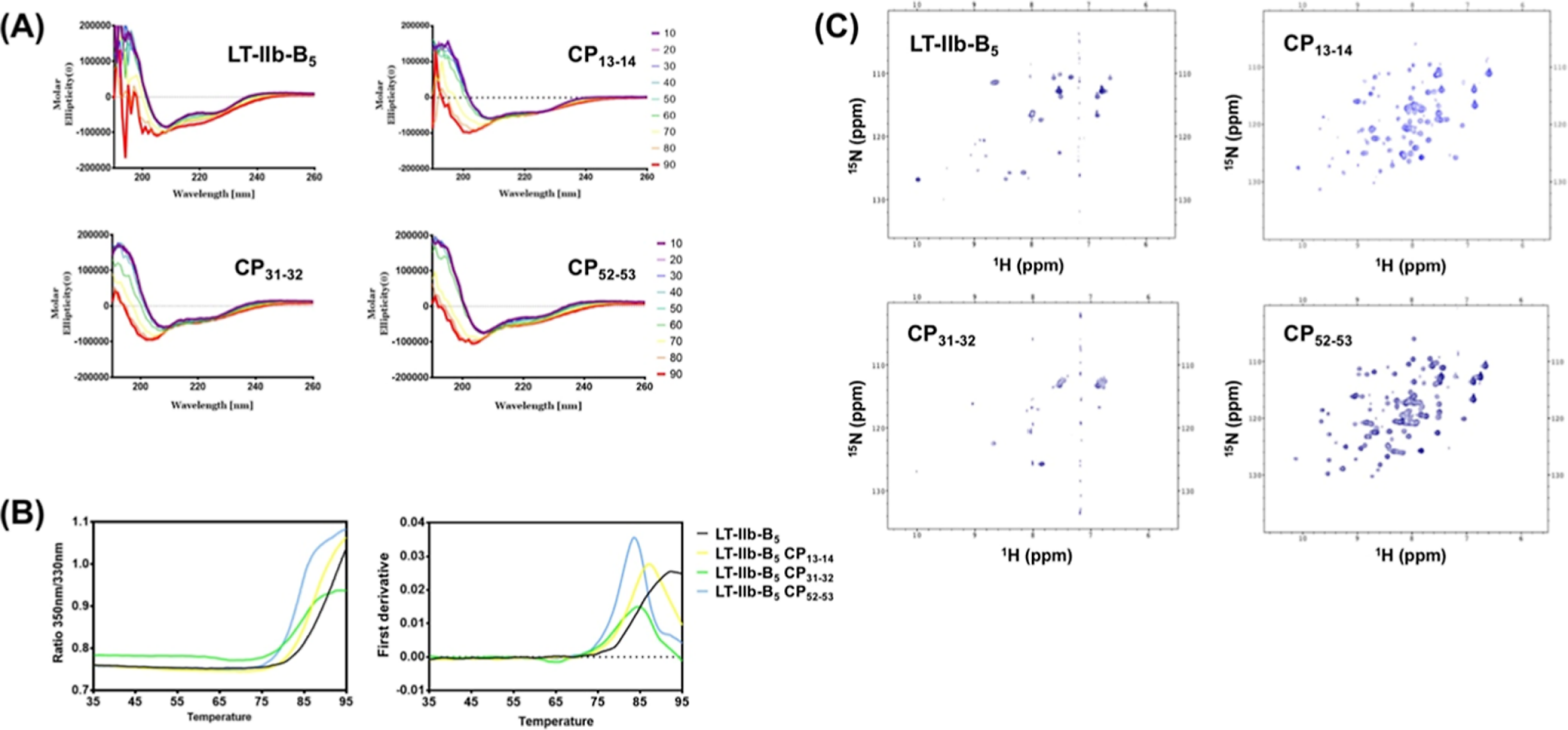
Structural stability of LT-IIb-B_5_ CPs. (A) CD spectra at different temperatures demonstrating LT-IIb-B_5_ CPs sharing the same secondary structural property as native protein. (B) Nano-DSF thermogram of LT-IIb-B_5_ CPs showing the fluorescence intensity ratio at 350 and 330 nm and the first derivatives of the fluorescence intensity ratio. (C) NMR ^1^H–^15^N HSQC spectra of LT-IIb-B_5_ CPs. The LT-IIb-B_5_ and CP_31–32_ show broadening resonances, indicating the backbone dynamics and aggregation property. The LT-IIb-B_5_ CP_13–14_ and CP_52–53_ show intense and dispersive resonances, indicating well-folded structure and better structural stability.

We checked the 2D ^1^H–^15^N HSQC spectra to realize the structural properties (Figure 2C). The HSQC spectrum of native LT-IIb-B_5_ showed relatively weak and broadened resonances. This phenomenon reflects the large molecular size (>50 kDa) and structural flexibility in solution. The spectrum was consistent with the precipitation of native LT-IIb-B_5_ in solution. In contrast, the HSQC spectrum of CP_13–14_ demonstrated strong and dispersive resonances. This represented a well-folded structure. Considering pentamer formation, the improvement in the spectral quality is most likely due to the reduced aggregation. The HSQC spectrum of CP_31–32_ showed weak signals similar to that of native LT-IIb-B_5_. Strong precipitation was also observed in CP_31–32_. This finding is consistent with the results of size-exclusion chromatography, which detected aggregated CP_31–32_ in solution (Figure 1E). Finally, CP_52–53_ exhibited the best HSQC spectrum among the constructs. Its signals were very intense and well-separated. The spectrum indicated a compact structure with less aggregation. Overall, relocating the N- and C-terminal ends to positions 13–14 and 52–53 results in well-folded LT-IIb-B_5_ CPs.

### The Binding of GD1a

We examined the binding of GD1a by 1D ^1^H NMR titration experiments. We titrated GD1a into solutions of CP_13–14_, CP_31–32,_ and CP_52–53_ (Figure 3A). In the test of CP_13–14_, the intensity of the GD1a peak did not change when an equal molar ratio of GD1a was titrated into the CP_13–14_ solution. Subsequent titrations showed an increase in the GD1a signal intensity, consistent with a drip multiple, indicating a lack of binding, even at higher GD1a concentrations. The same phenomenon was also observed in the test of CP_31–32_ (Figure 3A). Under the titration of an equal molar ratio of CP_31–32_, the intensity of the GD1a peak remained the same. As the GD1a ratio increased, the intensity of the GD1a peak increased proportionally. However, in the CP_52–53_ titration experiment, the peak of GD1a cannot be observed at the condition of a 1:1 molar ratio (Figure 3A). This is due to the intensity broadening when GD1a is in complex with a 50 kDa LT-IIb-B_5_ pentamer. The results demonstrated that CP_52–53_ retained the ability to bind to GD1a. The GD1a signal reappeared when excess GD1a was titrated. This also indicated that the binding stoichiometry is close to 1:1. To confirm the loss of GD1a-binding ability in CP_13–14_, we tested the HSQC spectra of ^15^N-labeled CP_13–14_ in the absence and presence of GD1a (Figure 3B). The spectra at 1:1 and 1:2 molar ratios showed no detectable perturbation. This result confirms the absence of interaction between GD1a and CP_13–14_. We skipped the test of the CP_31–32_ mutant because it did not produce a satisfactory HSQC spectrum.

**Figure 3:**
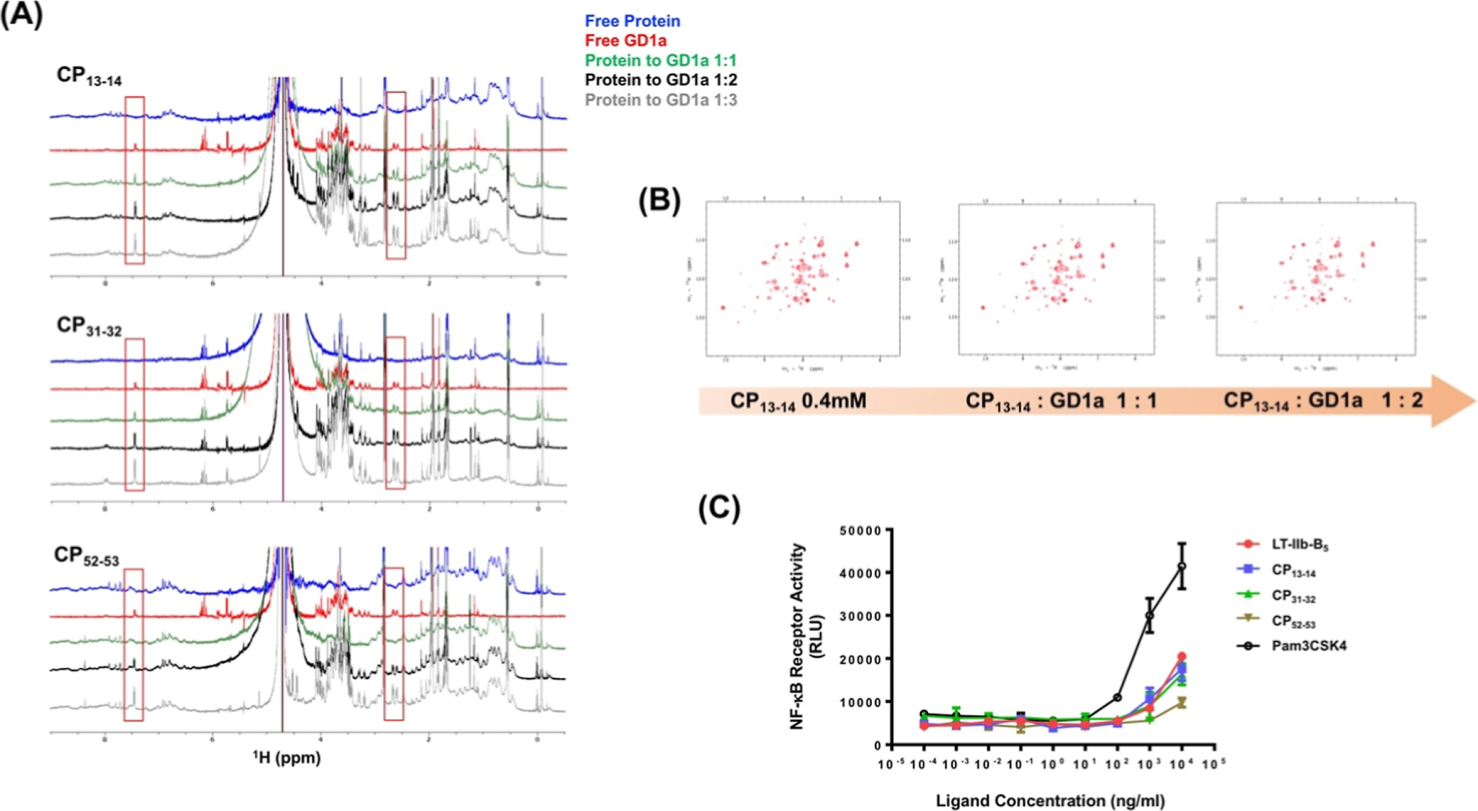
Receptor binding. (A) NMR 1D ^1^H spectra of GD1a titrated into CP_13–14_ solution, CP_31–32_ solution, and CP_52–53_ solution. Five ^1^H spectra are compared, that are spectra for free protein, free GD1a, protein to GD1a 1:1, 1:2, and 1:3. (B) ^1^H–^15^N HSQC spectra of ^15^N-labeled CP_13–14_ titrating with GD1a. The NMR spectra of free CP_13–14_ and CP_13–14_: GD1a with molar ratios of 1:1 and 1:2 are compared. The spectra were acquired in NMR buffer (50 mM Tris–HCl pH 7.0, 150 mM NaCl) at 298 K. (C) Human TLR-2/1 (hTLR-2/1) activity induced by the LT-IIb-B_5_ CPs. The binding between LT-IIb-B_5_ and hTLR-2/1 triggers the intracellular signaling pathway to activate NF-κB promoter, leading to the expression of the downstream luciferase gene. The luciferase activity is used to represent the activity of LT-IIb-B_5_ CPs toward activated TLR-2/1. Pam3CSK4 is a synthetic lipopeptide that activates the TLR-2/1, which is used as a positive control.

Of the three different CPs, positions 13–14 and 31–32 are the primary GD1a-binding sites for LT-IIb-B_5_. Using residues 13–14 and 31–32 as CP sites disrupts the peptide backbone connectivity, which, in turn, disrupts the integrity of the binding site and eliminates binding. In these two cases, the secondary binding site of loops 50–55 remains intact. The secondary binding site is still not effective for GD1a binding. Finally, the CP_52–53_ mutant retains the primary binding site and therefore preserves the ability to bind GD1a.

### hTLR-2/1 Activation

LT-IIb-B_5_ has been shown to activate human monocytes or mouse macrophages via the TLR-2/1 signaling pathway. We used the HEK 293A cells that were genetically transfected to overexpress the human TLR-2/1 heterodimer and NF-κB-driven luciferase. Treating the cells with different CPs revealed that the CPs retained the ability to activate the TLR-2/1 signaling pathway and trigger NF-κB-driven luciferase gene expression (Figure 3C). Pam3CSK4 was used as a positive control. CP_13–14_ and CP_31–32_ exhibited levels of TLR-2/1 activation similar to that of native LT-IIb-B_5_. CP_52–53_ has relatively low TLR-2/1 activity. Overall, the engineered CP_13–14_, CP_31–32_, and CP_52–53_ showed potential to enhance the immune response. However, CP_13–14_ and CP_31–32_ did not show GD1a-binding ability. The two CPs have a greater potential to be selected as protein adjuvants.

### Crystal Structures of CP_13–14_

The crystals of CP_13–14_ and CP_52–53_ were obtained with a high quality. However, CP_32–33_ never formed a crystal suitable for collecting X-ray diffraction data. The result is consistent with the structural instability of CP_32–33_. The structures of CP_13–14_ and CP_52–53_ had the resolution of 2.7 and 2.9 Å, respectively. The two structures of CP_13–14_ and CP_52–53_ were deposited in protein data bank (PDB 8GW2 and 8H2R).

The structure of CP_13–14_ was determined to be a pentamer. We observed the new terminal ends at residues Thr13 and Thr14 (Figure 4A). The electron density around the GSGS linker was weak and broken, reporting the dynamic local structure. The internal disulfide bond between Cys10 and Cys81 was observed. The overall structure of CP_13–14_ is very close to that of native LT-IIb-B_5_ (PDB 1QB5) (Figure 4B, left). We compared the secondary structures of residues 1–11 (α-helix) and 15–21 (β-strand), the portions adjacent to the new N- and C-termini (Figure 4B, right). There is no change to the backbone, and only slight changes occur in the side-chain orientation. Adding the GSGS linker and relocating the N- and C-termini did not affect the surrounding structural conformation. Notably, there is a structural change in loop 84–87. The local structure cannot be well overlapped with the native structure (Figure 4B, right).

**Figure 4:**
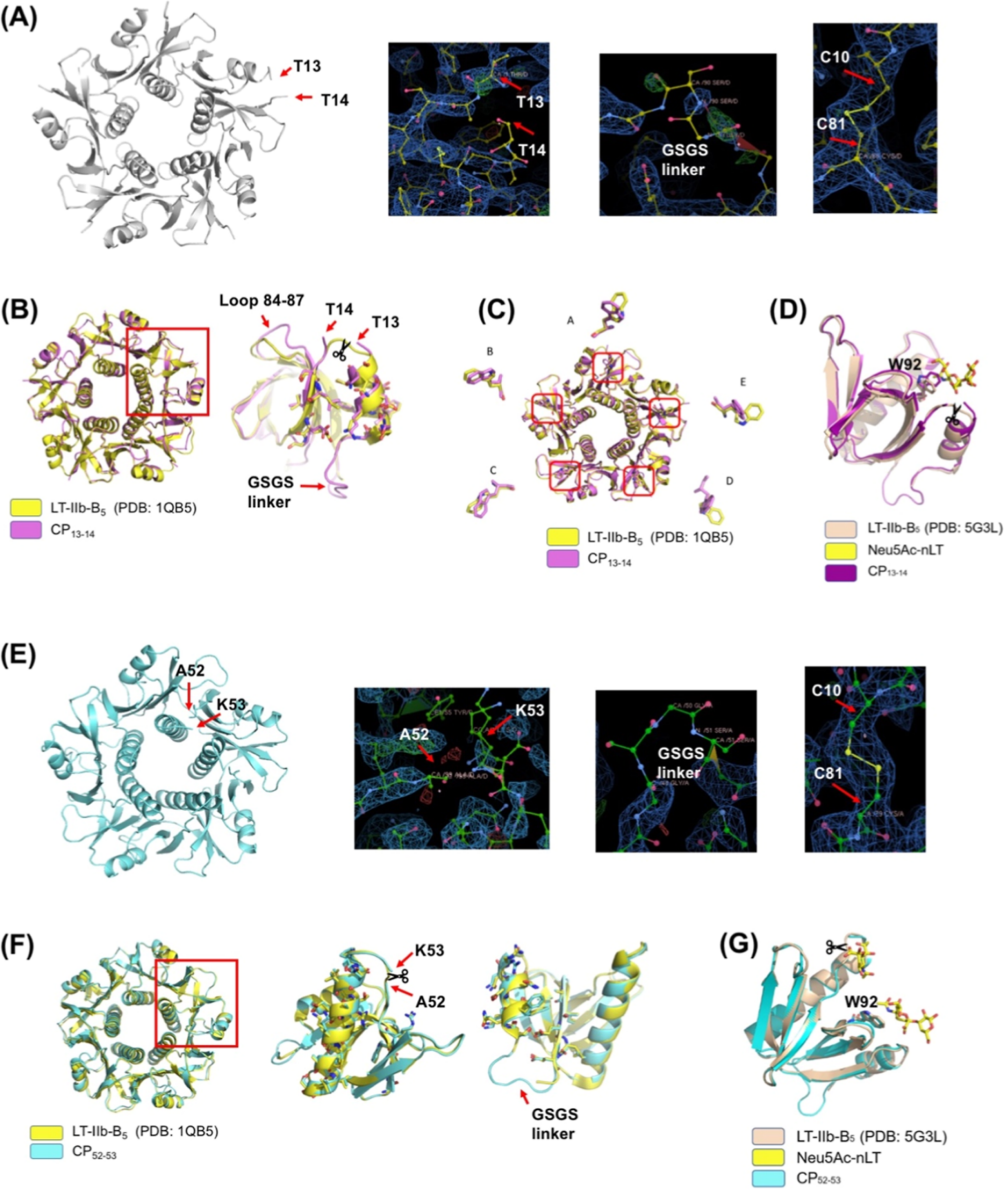
Crystal structure of CP_13–14_ and CP_52–53_. (A) Pentameric structure and partial electron density of CP_13–14_. Cartoon representation of the CP_13–14_ crystal structure at the resolution of 2.7 Å. The red arrows point to the N-terminal residue Thr14 and C-terminal residue Thr13. The electron density map of the N- and C-termini, the GSGS linker, and the disulfide bond between Cys10 and Cys 81 are depicted. (B) Overall structure of CP_13–14_ (magenta) and native LT-IIb-B_5_ (yellow, PDB 1QB5). The close-up view of the side-chain structure (sticks) near the position of the new N- and C-termini. (C) The close-up view of Trp92 side chain from each monomer. (D) Structural comparison of monomeric CP_13–14_ (purple) and LT-IIb-B_5_ in complexed with Neu5Ac-nLT (pink, PDB 5G3L). The side chain of Trp92 is shown in sticks. The position of Neu5Ac-nLT is located at the primary GD1a-binding site. (E) Pentameric structure and partial electron density of CP_52–53_. Cartoon representation of the CP_52–53_ crystal structure at the resolution of 2.9 Å. The red arrows point to the N-terminal residue Ala52 and C-terminal residue Lys53. The electron density map of the N- and C-termini, the GSGS linker, and the disulfide bond between Cys10 and Cys 81 are depicted. (F) Overall structure of CP_52–53_ (cyan) and native LT-IIb-B_5_ (yellow, PDB 1QB5). The close-up view of the side-chain structure (sticks) near the position of the new N- and C-termini. (G) Structural comparison of monomeric CP_52–53_ (cyan) and LT-IIb-B_5_ in complexed with Neu5Ac-nLT (pink, PDB 5G3L). The side chain of Trp92 is shown in sticks. The position of Neu5Ac-nLT is located at the primary and secondary GD1a-binding site.

Trp92 is a critical residue for GD1a binding. We compared the five Trp92 residues in the individual monomers. Not all Trp92 residues adopt the same side-chain orientation. Three of the residues (in units A, D, and E) underwent 30° to 90° rotations compared to the native structure (Figure 4C). However, the other Trp92 residues in units B and C have weak electron densities; therefore, their actual positions are unclear. This phenomenon implies the dynamic movement of the Trp92 side chain when positions 13 and 14 are disconnected. We then compared the CP_13–14_ crystal structure to the LT-IIb-B_5_ crystal structure in complex with Neu5Ac-nLT (PDB 5G3L) (Figure 4D). Although we observed a similar local structure around the primary GD1a-binding site, the new backbone opening at positions 13 and 14 disrupted the integrity of the binding site, and the rotating Trp92 side chain created an additional spatial barrier to hamper GD1a binding.

### Crystal Structures of CP_52–53_

In the case of the CP_52–53_ structure, the bond between Ala52 and Lys53 was disconnected (Figure 4E). Again, the dynamic GSGS linker had a weak electron density. The presence of the internal disulfide bond between Cys10 and Cys81 was also detected. We compared the crystal structures of CP_52–53_ and native LT-IIb-B_5_ (PDB 1QB5) (Figure 4F, left). The structures of native LT-IIb-B_5_ and CP_52–53_ are nearly identical. We particularly checked the secondary structures next to the new N- and C-termini. No substantial structural difference was observed (Figure 4F, right). We compared the crystal structure of CP_52–53_ with the crystal structure of LT-IIb-B_5_ in complex with Neu5Ac-nLT (PDB 5G3L) (Figure 4G). The primary GD1a-binding site remains intact, allowing for GD1a binding, and all Trp92 adopt the same side-chain orientation. The structure explains why CP_52–53_ retains the ability to bind to GD1a.

### Intranasal Immunization with N1 neuraminidase Protein Formulated with LT-IIb-B_5_ CPs Protein Adjuvant

To test the potential of CP_13–14_ and CP_31–32_ as protein adjuvants, CP_13–14_ and CP_31–32_ were, respectively, mixed with the mutated N1 NA protein (N1*) of the A/California/04/2009 (H1N1) virus strain (containing two mutation points N329*T*/K331T to increase cross-species activity) to prepare a nasal spray vaccine that 10 μg N1* proteins with 10 μg of LT-IIb-B_5_ protein adjuvant was used. Three nasal spray immunizations were conducted on mice at weeks 0, 3, and 6 (Figure 5A). Blood was collected at the eighth week to obtain the serum, and all mice were euthanized at the ninth week to obtain the BALF. ELISA was used to detect N1-specific IgG and IgA.

**Figure 5:**
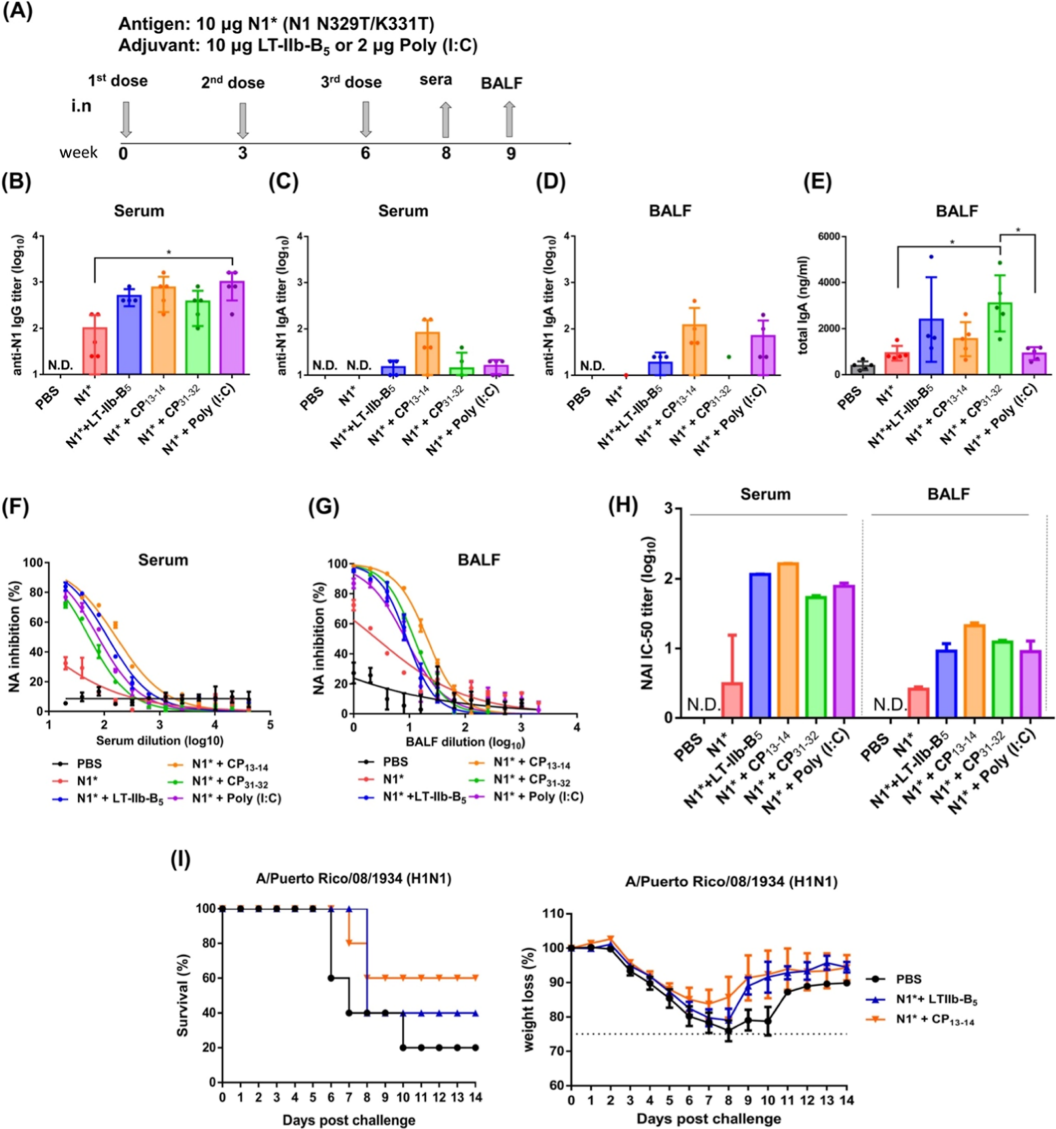
Evaluation of adjuvant efficacy of CP_13–14_ and CP_31–32_. (A) The schedule of intranasal immunization in the mouse model. Mouse experiments, collecting mouse serum and BALF. ELISA was used to detect NA-specific IgG and IgA. BALB/c mice (*n* = 5 per group) were treated with the NA recombinant protein of influenza strain N1 as the immune source, in which N1NA has two mutation sites (N329T and K331T), adjuvanted with LT-IIb-B_5_, CP_13–14_ or CP_31–32_, and intranasally immunized three doses. The polyinosinic acid-polycytidylic acid (poly­(I:C), a double-stranded RNA analog, was used as a positive control for the adjuvant since its ability to trigger antiviral immune responses. (B) N1NA-specific IgG titers in antisera, (C) N1NA-specific IgA titers in antisera, (D) N1NA-specific IgA titers in BALFs, and (E) total IgA titers in BALFs from each group of mice (*n* = 5 per group) and tested individually. These antibody titers among more than two comparable groups (excluding the PBS control) were analyzed using one-way ANOVA with Tukey’s multiple comparisons tests. Statistical significance in all results is indicated as follows: *p < 0.05 and **p < 0.01. Pooled antisera or BALFs of each immunized group of mice (*n* = 5) were measured for NA inhibition (NAI) against influenza strain A/California/04/2009 (H1N1) virus. ELLA was used to measure NA inhibition antibody titers as reductions in the NA enzyme activity of Pandemic Influenza A Virus (pH1N1 virus). NAI activity in (F) serum and in (G) BALF against A/California/04/2009 (H1N1) virus. (H) Corresponding half maximal inhibitory concentrations (IC_50_) are compared. The IC-50 NAI titers were obtained by fitting the dose-dependent curves from pooled sera from each immunized group using GraphPad Prism v6.01. (I) The survival rates and the body weight change against H1N1 virus. LT-IIb-B_5_ WT and CP_13–14_ adjuvants are respectively inoculated with N1NA recombinant protein. Three weeks after the final immunization, immunized mice (*n* = 5 per group) were infected with 5 × LD_50_ A/PR8 (H1N1) virus intranasally. Survival rates and body weights were monitored daily for 14 days postinfection. Survival curves were compared using the log-rank (Mantel–Cox) test. Body weight curves show the mean ± standard error of the percentage of body weight in surviving mice at each time point. The dashed line indicates the humane end point of the study, set at 75% of the initial body weight.

PBS and antigen (N1*) alone were used as the control group, and 2 μg of polyinosinic acid-polycytidylic acid (Poly­(I:C), a double-stranded RNA analog) was used as the positive control for the adjuvant since its ability to trigger antiviral immune responses.^32,33^ Whether in serum or BALF, the anti-N1 IgG and IgA titers of the adjuvanted group were significantly higher than those of the control group. CP_13–14_ produced more anti-N1 IgG and IgA in serum compared with LT-IIb-B_5_ and CP_31–32_ (Figure 5B,C), and it also produces more anti-N1 IgA in BALF (Figure 5D), while CP_31–32_ produces lower anti-N1 IgG and IgA responses. The p-values for serum IgG, serum IgA, and BALF IgA between CP_13–14_ and LT-IIb-B_5_ were 0.8652, 0.0848, and 0.3920, respectively. Anti-N1 IgA was not even detected in serum and BALF in the control group (Figure 5C,D). As for the total IgA content in BALF, CP_31–32_ produced the significant higher total IgA in the adjuvanted groups; instead, no significant enhancement was observed in LT-IIb-B_5_ and CP_13–14_ groups (Figure 5E).

To test the N1 NA inhibition (NAI) of the produced antibodies, an enzyme-linked lectin (agglutinin) assay (ELLA) was used to detect NA inhibition in each immunized group. Using the A/California/04/2009 (H1N1) virus as the virus strain, the inhibition derived from the activity between N1 protein and vaccination-induced antibodies were recorded. By the series of dilutions, NAI of serum (Figure 5F) and BALF (Figure 5G) were gradually reduced. The corresponding half-maximal inhibitory concentrations (IC_50_) were calculated (Figure 5H). It was found that the serum group with adjuvants had a certain NAI ability even when diluted to 100× which is significantly higher than that of the control group. Moreover, CP_13–14_ had a relatively high IC_50_ titer. The BALF group showed the same trend. CP_31–32_ had comparable IC_50_ titer to native LT-IIb-B_5_ in the BALF group and lower tier in the serum group (Figure 5H). This experiment proves that among the three adjuvants, CP_13–14_ produced the most potent antibodies to inhibit NA enzyme activity.

N1* protein with CP_13–14_ adjuvant was found to have the highest NAI titer. In the protective immunity mouse experiment, we tested the protective immunity against virus challenge infection and compared it to the case of LT-IIb-B_5_ (Figure 5I). Three weeks after the final immunization, immunized mice (*n* = 5 per group) were infected with 5 × LD_50_ A/PR8 (H1N1) virus intranasally. Survival rates and body weights were monitored daily for 14 days after the infection. The results showed that N1* combined with either CP_13–14_ or LT-IIb-B_5_ adjuvant conferred the protection against H1N1 virus infection. CP_13–14_ showed better protective immunity than native LT-IIb-B_5_, and the survival rates were 60% vs 40%, respectively.

### Intranasal Immunization with Trivalent NA Proteins Formulated with the LT-IIb-B_5_ CPs Protein Adjuvant

To refocus the antibody response to cross-reactive NA epitopes, we further evaluated the effect of CP_13–14_ by using trivalent NA proteins. Three recombinant NA proteins of N1*, N2 and BN from A/California/04/2009 (H1N1), A/Udorn/307/1972 (H3N2), and B/Darwin/07/2019 (IBV) were used in the intranasal immunization at three different dosages: 5 μg of each protein (15 μg total), 7 μg of each protein (21 μg total), and 10 μg of each protein (30 μg total), along with 10 μg of CP_13–14_ protein adjuvant. The first group, receiving total 15 μg of NA antigens, was also administered 2 μg of poly­(I:C) as a positive control. With the same nasal spray immunization procedure (Figure 6A), the results indicated that anti-N1 and anti-N2 IgG titers in the antisera increased as the trivalent NA dosage rose from 15 μg to 21 μg and 30 μg, while anti-BN IgG titers remained consistently high across all three dosages (Figure 6B). Similar trends were observed for anti-N1, anti-N2, and anti-BN IgA titers in the antisera (Figure 6C). The titers of NAI antibodies in the antisera also showed a dose-dependent increase for the trivalent NA dosages (from 15 to 21 and 30 μg) with the CP_13–14_ adjuvant, specifically against H1N1 and H3N2, while the NAI titers against IBV remained consistently high at all dosages (Figure 6D). Antisera viral replication inhibition titers showed that titers against H1N1 and H3N2 viruses remained in similar ranges across all dosages. However, the titers against IBV for the trivalent NA dosages peaked at 21 and 30 μg when combined with CP_13–14_ (Figure 6E). We also measured IgG1 and IgG2a subtypes in the antisera, revealing that IgG2a to IgG1 ratios for all three dosage groups were between 0.2 and 0.6, compared to 0.6 to 1.0 for the group of 15 μg of antigens with the poly­(I:C) adjuvant (Figure 6F). The IgG2a/IgG1 ratio is a key indicator of the type of T-helper (Th) immune response. A ratio greater than 1 suggests an effective cell-mediated Th1 response against viruses and bacteria. A ratio of less than 1 suggests a humoral Th2 response, which is effective against parasites and allergies. We found that LT-IIb-B_5_ components promote stronger Th2 responses and poly­(I:C) exhibited a relatively balanced Th1/Th2 response. However, poly­(I:C) is generally considered to be Th1-biased.^34^ This evaluation needs to consider the effect from the mouse strain. BALB/c mice have been reported to exhibit an intrinsic Th2-biased immune profile compared with other commonly used strains.^35^ The Th2 skewing was partially attributed to the measurement. Thus, LT-IIb-B_5_ likely elicits a more balanced Th1/Th2 response.

**Figure 6:**
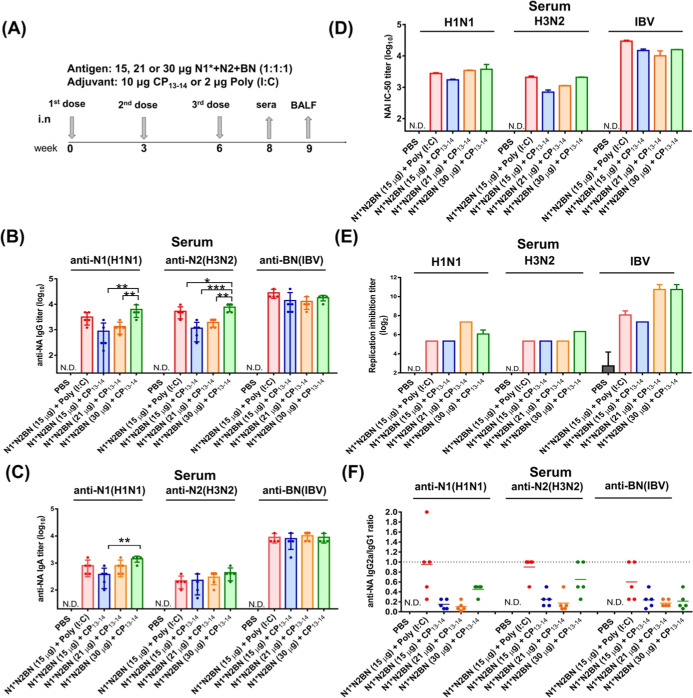
Serum antibody response in vaccinated mice. (A) Immunization scheme. BALB/c mice (*n* = 5) were intranasally vaccinated in a prime/boost/boost regimen with different doses (15, 21, or 30 μg) trivalent NAs protein (N1* + N2 + BN) + 10 μg CP_13–14_ or 2 μg poly­(I:C) as a control. Serum samples were collected after 2 weeks of the third vaccination. NA-specific (B) IgG and (C) IgA titers in antisera of each group (*n* = 5) were measured by ELISA assay. The IgG and IgA antibody titers among more than two comparable groups (excluding the PBS control) were analyzed using one-way ANOVA with Tukey’s multiple comparisons tests. Statistical significance in all results is indicated as follows: *P < 0.05; **P < 0.01; ***P < 0.001. NA functional antibody titers in post-third dose antisera pooled from each immunized groups of mice were determined in (D) ELLA and (E) replication inhibition assay against H1N1 (A/California//07/2009), H3N2 (A/Udorn/307/1972), and IBV (B/Darwin/07/2019) viruses. The IC_50_ NAI titers were obtained by fitting the dose-dependent curves from pooled sera from each immunized group using GraphPad Prism v6.01. The IC_50_ values of the NAI assay represent the 50% reduction of virus enzyme activity. Virus growth inhibition ability in sera was quantified by hemagglutination assay. (F) The IgG2a/IgG1 ratio was evaluated by ELISA assay. Data showed the mean ± SD. Statistical analyses were conducted by one-way ANOVA with Tukey’s multiple comparison test (*P < 0.05; **P < 0.01; ***P < 0.001).

To further characterize the mucosal responses, BALF samples were collected from all groups 3 weeks after the third immunization dose. These samples were analyzed for anti-NA IgA and NAI antibody titers as well as viral replication inhibition. The results showed dose-dependent increases in anti-N1 and anti-N2 IgA antibodies in BALFs against H1N1 and H3N2 viruses, while titers against IBV remained consistently high across the total dosages of 15 μg, 21 μg, and 30 μg (Figure 7A,B). Viral replication inhibition from the BALF samples was not significant at all dosages for H1N1 and H3N2 but significantly effective in inhibiting IBV in the 21 and 30 μg dosage groups (Figure 7C).

**Figure 7:**
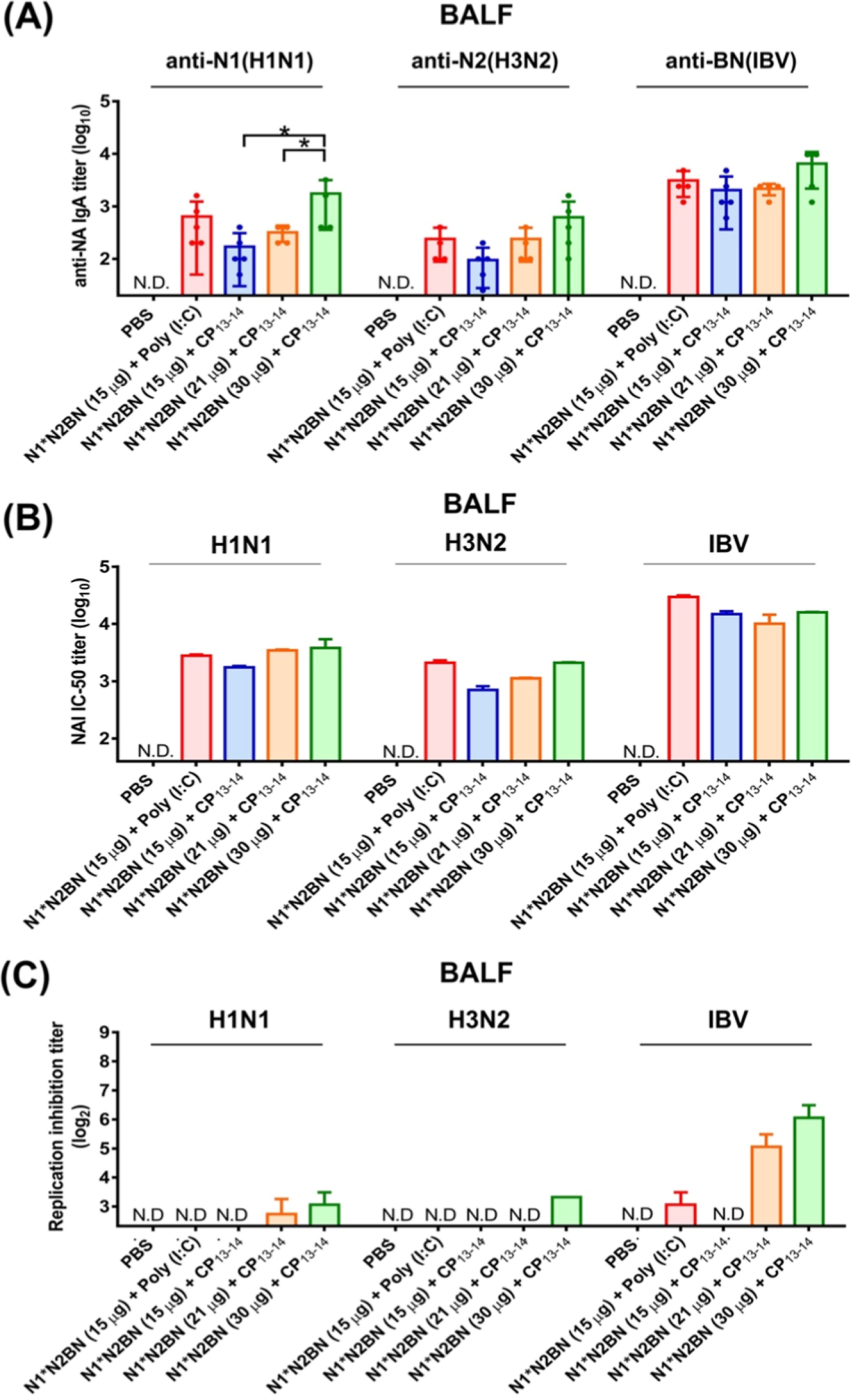
BALF antibody response in vaccinated mice. (A) Anti-NA immune response at the mucosal site following the three inoculation. After 3 weeks of the third vaccination, mice were humanely euthanized and BALF was collected. Anti-NA IgA levels in BALFs were determined by ELISA assay of each group (*n* = 5) individually. (B) IC_50_ values of NA inhibition antibody titers against H1N1 (A/California//07/2009), H3N2 (A/Udorn/307/1972), and IBV (B/Darwin/07/2019) were quantified by ELLA from the pooled samples from each immunized groups of mice. (C) Virus growth inhibition efficacy of BALF samples was measured by replication inhibition assay from the pooled samples from each immunized groups of mice. The end points were determined by hemagglutination assay. Data showed the mean ± SD. Statistical significance was analyzed by one-way ANOVA with Tukey’s multiple comparison test (*P < 0.05; **P < 0.01; ***P < 0.001).

Finally, we assessed the cross-protective immunities induced by above-mentioned vaccine candidates formulated with the protein adjuvant in the mouse model. After intranasal immunization with three doses of trivalent protein (30 μg N1* + N2 + BN) formulated with LT-IIb-B_5_ CP_13–14_, all mice were challenged with 5-fold greater than the 50% murine LD50 of either H1N1 (A/Puerto Rico/8/1934) or H3N2 (A/Aichi/2/1968) virus. Our results demonstrated that the trivalent nasal spray vaccine conferred complete protection against heterologous H1N1 and H3N2 viral challenge infections. In contrast, the PBS control group succumbed to virus infections, with survival rates of 20% and 0%, respectively (Figure 8A,C). Moreover, the trivalent vaccinated group also showed more efficient body weight recovery than the PBS control group, starting from day 8 (for H1N1, Figure 8B) and day 4 (for H3N2, Figure 8D) postinfection.

**Figure 8:**
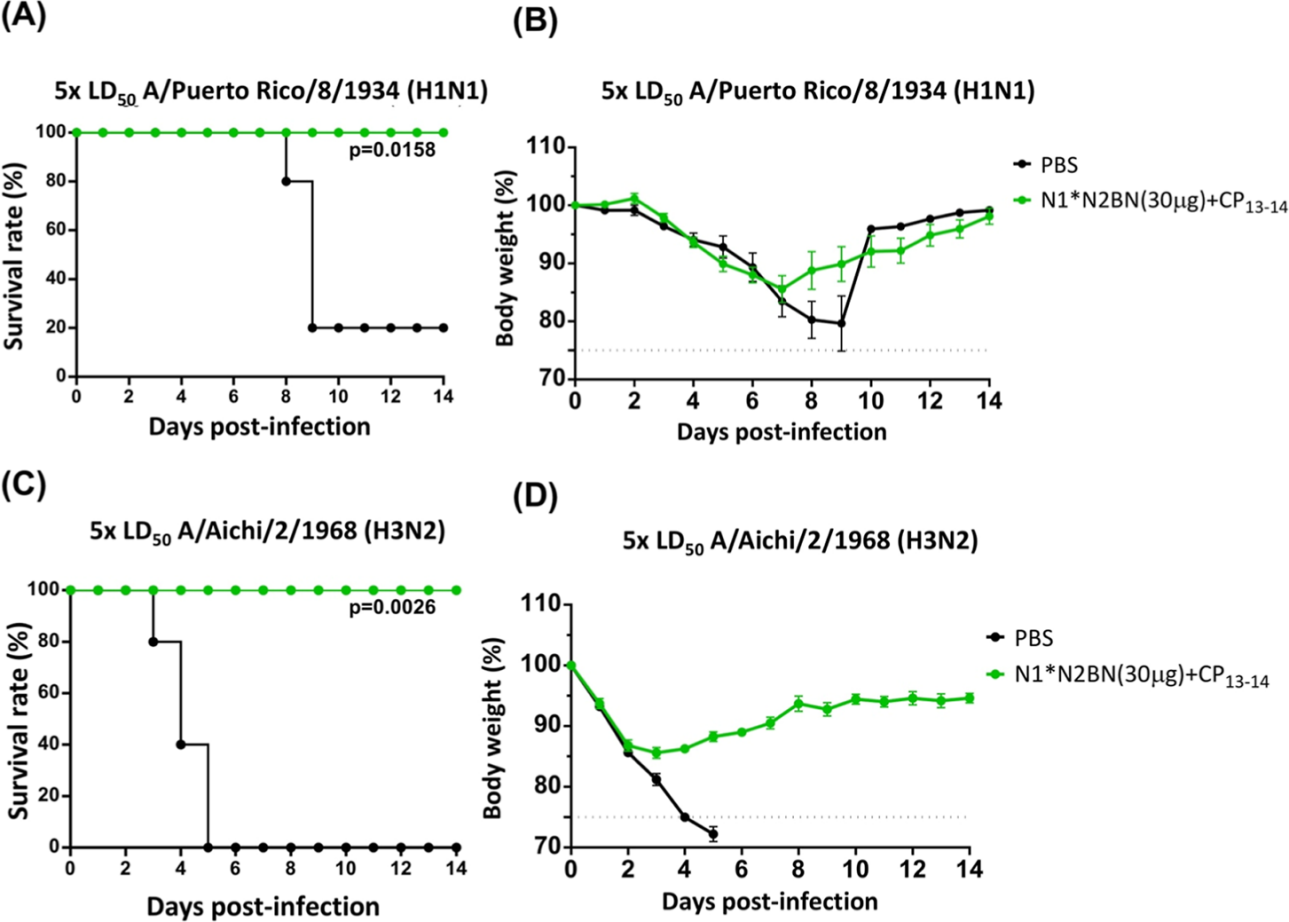
Efficacy evaluation of trivalent influenza vaccine in mice. 6 Week-old BALB/c female mice were intranasally inoculated with trivalent (N1* + N2 + BN) influenza vaccines formulated with LT-IIb-B_5_ CP_13–14_ adjuvants at three-week intervals. PBS-immunized group was also included as a negative control. After three doses of immunization, the mice were then, respectively, challenged with 5× LD_50_ H1N1­(A/P Puerto Rico/8/1934) or H3N2 (A/Aichi/2/1968) virus and daily monitored for 14 days. (A,C) The survival rates and (B,D) the body weight changes of each immunized group were calculated. The mean ± standard error of the mean of body weight at each group was indicated. The dash line means the humane end point of the study. Survival curves were analyzed using the log-rank (Mantel–Cox) test, with statistical significance defined by *P* values.

## Discussion

Nasal administration of vaccines provides a primary defense and generates antigen-specific mucosal immunity, as well as systemic immunity to foreign antigens. Antigen can be recognized in lymphoid tissues associated with the nasopharynx to induce mucosal immune responses. Nasal delivery of vaccines requires the use of adjuvants to induce effective mucosal immunity.^36,37^ The ADP-ribosylating enterotoxins for CT and *E. coli* heat-labile enterotoxin (LT) are still recognized as the most potent mucosal adjuvants. Here, we used the CP strategy to design the pentameric B subunit of LT-IIb to be a novel protein adjuvant. We relocated protein N- and C-terminal ends to remove GD1a-binding ability but reserve the TLR-2/1 binding. The backbone disruption near the GD1a-binding site disrupted the integrity of the GD1a-binding site. The rest of the LT-IIb-B5 structure remained unchanged. Therefore, no attenuation of the TLR-2/1 receptor interaction was observed, while similar NF-κB activity was observed. In the study, we presented the case of LT-IIb-B_5_ CP_13–14_ and identified the LT-IIb-B_5_ CP_13–14_ to be the potent mucosal adjuvant candidate through functional evaluation. Compared to the protein adjuvant using CT or LT hexameric AB_5_ structure, the current design brought an advantage in safety. Without the GD1a-binding ability, we expect that protein internalization is eliminated. Even if an LT A unit is obtained from an infection, the LT-IIb-B_5_ CP_13–14_ will not be able to cause toxin internalization. This would prevent the side effect derived from LT toxicity. That would greatly reduce the concern about developing LT-IIb-B_5_ as a protein adjuvant.

The strategy of CP further provides other advantages. First, the circularly permuted CP_13–14_ showed increased stability due to the reduced conformational flexibility. Thus, the CP_13–14_ variant retained better structural properties in solution. Even with a slightly lower *T*_m_, we observed less aggregation in the NMR experiment. The property gives advantage that makes proteins active over a wider range of temperatures and concentrations to improve the utility in bioindustry. CP_13–14_ maintains proper protein folding by minimizing unfavorable intermolecular aggregates, which occurs significantly in native LT-IIb-B_5_. Second, the sequence of LT-IIb-B_5_ CP_13–14_ is almost identical to that of the native protein, except for the substitution of the backbone disconnection in the different position. Together with the exact same structure, the induced immunity could be close to the native protein. The potency found in LT-IIb-B_5_ was transferred to the CP variant.

For influenza vaccine development, influenza A virus remains a persistent threat to global health because of continuous antigen drift and shift of two major surface glycoproteins, hemeagglutinin (HA) and NA.^38,39^ Compared to HA, NA has less antigenic drift,^40,41^ and the enzymatic active site of NA is highly conserved among different subtypes and represents a compelling target for universal vaccine design. In humans, the elicitation of NAI antibodies has been demonstrated through natural infection or vaccination.^42^ The levels of NAI antibodies have been shown to correlate with protection,^43,44^ reduce viral replication and shedding,^45^ and inversely correlate with illness severity, symptoms, and disease duration.^46^ In this study, we demonstrated that intranasal immunization of mice with trivalent NA proteins formulated with the CP_13–14_ adjuvant elicited potent NAI antibodies in both antisera and BALF against H1N1, H3N2, and influenza B viruses. Additionally, this immunization conferred protection against H1N1 and H3N2 virus challenges. Although we did not assess T-cell responses in the spleen, nasal-associated lymphoid tissues, or lungs in this study, the IgG2a/IgG1 ratio in antisera suggests that the trivalent NA proteins formulated with the CP_13–14_ adjuvant predominantly elicited a Th2-type immune response. Nevertheless, it is likely that protection was also mediated by T-cell responses, as a recent study demonstrated that intranasal vaccination with NA and M2e virus-like particles induced not only high titers of IgA antibodies but also robust CD4^+^ and CD8^+^ T-cell responses, germinal center B cells, plasma cells, and early innate immune activation in mucosal tissues—resulting in broader protection than that achieved by intramuscular immunization.^47^

Based on the success of the influenza mucosal vaccine in mice, clinical trials in humans could be considered. Prior to this, we expect a preclinical-to-clinical translation to use a ferret model. The ferret model is the gold standard for the influenza animal model. Due to emulation of clinical features associated with human disease and similar physiological/anatomical features, the safety, toxicity, and efficacy of the vaccine/adjuvant formulations, along with the dose-finding, will be validated in the preclinical ferret model to ensure they are best adapted for use in humans. This evaluation is required to assess the protection conferred by the trivalent NA proteins with the CP_13–14_ adjuvant against virus transmission via the respiratory tract. Once safety and immunogenicity were confirmed, we proceeded to phase I trials in humans. While our study used a three-dose intranasal regimen to maximize mucosal uptake in a mouse model, applying this to humans requires careful consideration of the frequency of dosing and the volume of delivery. In a clinical setting, this regimen could be adapted into a well-planned dosing schedule such as a daily dosing schedule over 3 days to maintain therapeutic concentrations while minimizing local mucosal irritation. Furthermore, using precision intranasal delivery devices that target the olfactory epithelium rather than standard nasal sprays might be essential in this experimental design.

## Conclusions

We identified a potent CP candidate of LT-IIb-B_5_ variants with a backbone opening at positions 13–14 (CP_13–14_). The CP pentamer lost its ability to bind to GD1a, yet its ability to activate TLR-2/1 is maintained. In the intranasal vaccination using trivalent NA proteins formulated with LT-IIb-B_5_ CP_13–14_, we demonstrated the complete protections against heterologous H1N1 and H3N2 viral challenge infections. This study purposes a new design for LT-IIb-B_5_ protein adjuvant and addresses the urgent need for a novel, efficient, and safer mucosal adjuvant for respiratory diseases.

## Supplementary Material



## Notes

Prediction of LT-IIb-B_5_ CP sites; sequences of the related LT-IIb-B_5_ constructs; and X-ray data collection and refinement statistics of the structures of CP_13–14_ and CP_52–53_ (PDF)
