# Supplementary material for: Circular Permutation of the E. coli Heat-Labile Enterotoxin Pentameric B Subunit for Mucosal Vaccine Adjuvant Design
Source: ACS Omega. 2026 Jan 25;11(5):8784–98. doi: 10.1021/acsomega.5c12660 (PMC12903156; doi:10.1021/acsomega.5c12660)
Supplement: Supplementary file 1 [file ao5c12660_si_001.pdf]

# Supporting Information

# Circular permutation of the *E. coli* heat-labile enterotoxin pentameric B subunit for mucosal vaccine adjuvant design

Sheng-Han Hsu[a], Da-You Xie[b], Cheng-Yen Tsai[a], Wei-Shuo Lin[b], Chu-Ya Wu[c], Yi-Hung Lin[d], Wen-Chun Liu[e], Suh-Chin Wu[b*], Shih-Che Sue[a*]

[a]*Institute of Bioinformatics and Structural Biology, National Tsing Hua University, 101, Section 2, Kuang-Fu Road, Hsinchu 300044, Taiwan*

[b]*Institute of Biotechnology, National Tsing Hua University, 101, Section 2, Kuang-Fu Road, Hsinchu 300044, Taiwan*

[c]*Instrumentation Center, National Tsing Hua University, 101, Section 2, Kuang-Fu Road, Hsinchu 300044, Taiwan*

[d]*National Synchrotron Radiation Research Center, 101 Hsin-Ann Road, Hsinchu Science Park, Hsinchu 300092, Taiwan*

[e]*Biomedical Translation Research Center, Academia Sinica, 128 Section 2, Academia Road, Nankang, Taipei 115201, Taiwan*

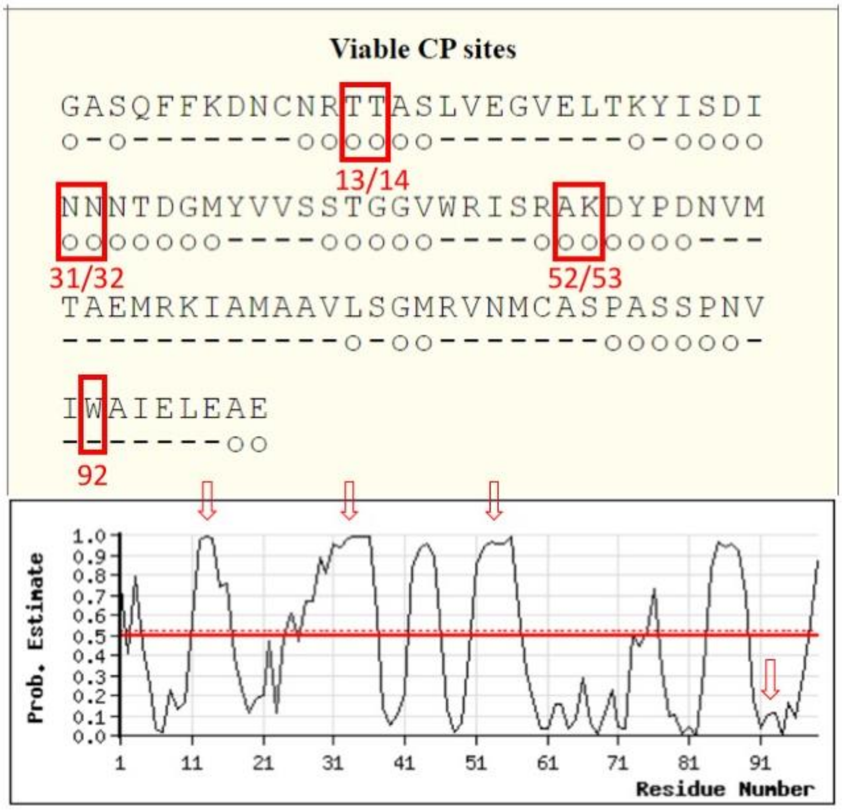


**Figure S1.** The prediction of LT-IIb-B5 circular permutation (CP) sites by CPred program. The sites involved in GD-1a binding are boxed and the related CP predictions are marked in the estimation (below) by arrows. The higher CP score represents the better potency to be selected as a CP site.

LT-IIb-$B_5$

GASQFFKDNCNRTTASLVEGVELTKYISDINNNTDGMYVVSSTGGVWRISRAKDYPDNVMTAEMRKIA
MAAVLSGMRVNMCASPASSPNVIWAIELEAEHHHHHH

$CP_{13\text{-}14}$

$^{14}$TASLVEGVELTKYISDINNNTDGMYVVSSTGGVWRISRAKDYPDNVMTAEMRKIAMAAVLSGMRVN
MCASPASSPNVIWAIELEAEGSGSGASQFFKDNCNRT$^{13}$HHHHHH

$CP_{31\text{-}32}$

$^{32}$NNTDGMYVVSSTGGVWRISRAKDYPDNVMTAEMRKIAMAAVLSGMRVNMCASPASSPNVIWAIEL
EAEGSGSGASQFFKDNCNRTTASLVEGVELTKYISDIN$^{31}$HHHHHH

$CP_{52\text{-}53}$

$^{53}$KDYPDNVMTAEMRKIAMAAVLSGMRVNMCASPASSPNVIWAIELEAEGSGSGASQFFKDNCNRTTA
SLVEGVELTKYISDINNNTDGMYVVSSTGGVWRISRA$^{52}$HHHHHH

**Figure S2.** The sequences of LT-IIb-$B_5$ and the related CP constructs.

**Table S1.** X-ray data collection and refinement statistics of the structures of $CP_{13-14}$ and $CP_{52-53}$.

| **Crystal** | **$CP_{13-14}$** (PDB 8GW2) | **$CP_{52-53}$** (PDB 8H2R) |
|---|---|---|
| **Data collection** | | |
| Space group | C121 | C121 |
| Cell dimension a, b, c (Å) | 70.43, 69.54, 107.48 | 74.38, 69.78, 88.33 |
| Wavelength | 1.00000 | 1.00000 |
| Resolution (Å)* | 30.00-2.70 (2.91-2.80) | 50.00-2.9 (3.00-2.95) |
| CC½ (%)* | 84.1 | 87.8 |
| $R_{meas}$ (%)* | 14.8 (64.5) | 17.8 (63.8) |
| $R_{merge}$ (%)* | 12 | 14.17 |
| I/σ* | 12.1 (2.3) | 8.1 (1.7) |
| Completeness* (%)* | 96.3 (98.9) | 97.8 (90.7) |
| Redundancy* | 2.8 (3.1) | 3.3 (2.9) |
| **Refinement** | | |
| Resolution range (Å) | 2.71 | 2.9 |
| No. reflections | 13472 | 7526 |
| $R_{work}$(%)/$R_{free}$(%) | 20.29/28.65 | 22.53/35.02 |
| No. atom | 3784 | 3845 |
| Macromolecules | 3745 (515 residues) | 3845 (515 residues) |
| Solvent | 39 | 0 |
| B-factors (Å$^2$) | 40.74 | 49.82 |
| R.M.S deviations | | |
| Bond length (Å) | 0.008 | 0.009 |
| Bond angle (°) | 1.04 | 1.24 |
